# p53-mediated redox control promotes liver regeneration and maintains liver function in response to CCl_4_

**DOI:** 10.1038/s41418-021-00871-3

**Published:** 2021-10-09

**Authors:** Timothy J. Humpton, Holly Hall, Christos Kiourtis, Colin Nixon, William Clark, Ann Hedley, Robin Shaw, Thomas G. Bird, Karen Blyth, Karen H. Vousden

**Affiliations:** 1 The Francis Crick Institute, London, NW1 1AT UK; 2 Cancer Research UK Beatson Institute, Glasgow, G61 1BD UK; 3 Institute of Cancer Sciences, University of Glasgow, Glasgow, G61 1QH UK; 4 MRC Centre for Inflammation Research, The Queen’s Medical Research Institute, University of Edinburgh, Edinburgh, EH16 4TJ UK

**Keywords:** Tumour-suppressor proteins, Physiology

## Abstract

The p53 transcription factor coordinates wide-ranging responses to stress that contribute to its function as a tumour suppressor. The responses to p53 induction are complex and range from mediating the elimination of stressed or damaged cells to promoting survival and repair. These activities of p53 can modulate tumour development but may also play a role in pathological responses to stress such as tissue damage and repair. Using a p53 reporter mouse, we have previously detected strong induction of p53 activity in the liver of mice treated with the hepatotoxin carbon tetrachloride (CCl_4_). Here, we show that p53 functions to support repair and recovery from CCl_4_-mediated liver damage, control reactive oxygen species (ROS) and limit the development of hepatocellular carcinoma (HCC), in part through the activation of a detoxification cytochrome P450, CYP2A5 (CYP2A6 in humans). Our work demonstrates an important role for p53-mediated redox control in facilitating the hepatic regenerative response after damage and identifies CYP2A5/CYP2A6 as a mediator of this pathway with potential prognostic utility in human HCC.

## Introduction

The TP53 (p53) transcription factor coordinates diverse aspects of the cellular stress response and is capable of engaging both pro-survival and pro-death pathways [1]. Although p53 was initially identified through its association with cancer, it also has broader roles in organismal health. p53 is required for efficient implantation of embryos into the uterus [2], promotes stamina during exercise [3, 4], limits fibrosis after chronic liver injury [5], and protects against *Listeria monocytogenes* infection [6]. Conversely, unrestrained activation of p53 in embryos is rapidly lethal [7–9], p53 promotes B-cell apoptosis in type 2 diabetes mellitus [10], and in ischaemia, inhibition of p53 is protective [11–13]. These disparate outcomes suggest a nuanced balance between divergent aspects of p53 activity.

The liver provides an excellent setting in which to examine the intersection of p53 signalling in cancer and normal biology. Disruption of *TP53* is observed in 30–40% of human hepatocellular carcinomas (HCCs) [14], suggesting tumour-suppressive functions for p53 in the liver. Indeed, the loss of hepatic p53 alone is sufficient to promote liver cancer in mice, albeit at long latency [15]. Even so, wild-type p53 is retained in more than half of human HCCs, with a previously identified *TP53* gene expression signature characterising this group [14]. These observations suggest that aspects of p53 function may also support—or at least not directly antagonise—hepatic tumourigenesis.

Although the liver is largely quiescent in adults, it can undergo rapid regeneration after damage or resection [16]. As in HCC, the p53 pathway has been reported to support and antagonise liver regeneration. It has been shown that p53 limits liver damage after acetaminophen overdose and protects mitotic fidelity after partial hepatectomy, indicating protective roles for p53 in the hepatic injury response [17–19]. Similarly, p53-deficient mice exhibit enhanced sensitivity to high-dose irradiation during CCl_4_-induced regeneration [20]. In addition, the loss of CDKN1A/p21, a p53 target, has been reported to impair liver regeneration in certain liver damage models [21, 22]. However, robust activation of p21 has also been shown to promote senescence and to limit the regenerative response, and p21 loss can allow for survival after severe liver damage—suggesting that this arm of the p53 response may also impede liver regeneration [21, 23]. More directly, unrestrained activation of p53 is lethal in hepatocytes, p53-mediated apoptosis contributes to disease progression in a model of non-alcoholic steatohepatitis, and p53 activity promotes fibrosis in a model of chronic regeneration in rats [24–27]. Thus, the role of p53 in the hepatic response to toxic damage is not clear and may be dependent on the nature and severity of the initiating damage.

Here, we utilise CCl_4_-mediated liver regeneration as a model system to investigate the function of p53 in liver biology. Our work demonstrates a role for p53-mediated redox control in facilitating the hepatic regenerative response after damage. We identified CYP2A5/CYP2A6 as a mediator of this pathway with potential prognostic utility in human HCC.

## Results

### Liver-specific loss of p53 exacerbates liver damage and increases ROS during CCl_4_-mediated liver regeneration

**Fig. 1:**
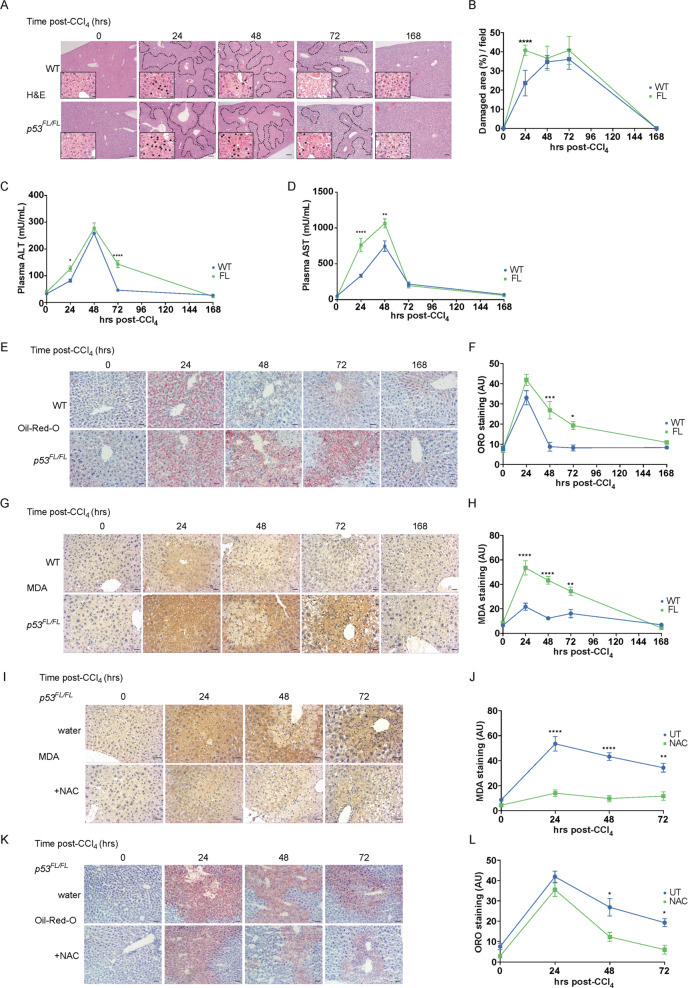
Loss of liver p53 exacerbates liver damage and increases ROS during CCl4-mediated liver regeneration. Representative H&E images (**A**) and quantification (**B**) of damaged area (% per field) in livers from *Albumin*-*Cre*; *p53*^WT/WT^ mice (WT) and *Albumin*-*Cre*; *p53*^FL/FL^ (FL) mice at indicated times (hours) following CCl_4_ treatment. Damaged areas outlined. Scale bars 100 μm. Higher magnification inset images highlight centrilobular liver damage including vacuolisation (black arrows). These images are taken from different H&E slides than those depicted for damaged area. Scale bars 10 μm. Images reproduced without annotation and at full size in Fig. S1 C/D. Quantifications from *N* = 2 untreated (0 h) mice/group, *N* = 6 mice/group at 24 h, *N* = 5 WT and *N* = 4 FL mice at 48 h, *N* = 7 mice/group at 72 h, N = 3 mice/group at 168 h. Data presented as mean ± SEM and analysed using two-way ANOVA with Holm-Sidak’s multiple comparisons test and multiplicity-adjusted *p* values. ****p < 0.0001. Plasma ALT (**C**) and AST (**D**) activity (mU/mL) in *Albumin-Cre; p53*^*WT/WT*^ (WT) and *Albumin-Cre; p53*^*FL/FL*^ (FL) mice treated as in (**A**). *N* = 3 untreated (0 h) mice/group. *N* = 4 mice/group/time point thereafter. Each data point represents the mean from technical duplicates per mouse. Data presented as mean ± SEM and analysed using two-way ANOVA with Holm-Sidak’s multiple comparisons test and multiplicity-adjusted p-values. *p < 0.05, **p < 0.01, ****p < 0.0001. Staining (**E**) and quantification of stain area (**F**) of frozen sections for oil-red-O (ORO) in *Albumin-Cre; p53*^*WT/WT*^ (WT) and *Albumin-Cre; p53*^*FL/FL*^ (FL) mice treated as in (**A**). Scale bars 20 μm. Quantification from *N* = 5 untreated (0 h), 24 h, and 48 h mice/group, *N* = 7 mice/group at 72 h, and N = 3 mice/group at 168 h. Data presented as mean ± SEM and analysed using two-way ANOVA with Holm-Sidak’s multiple comparisons test and multiplicity-adjusted *p* values. *p < 0.05, ***p < 0.001. Data from *p53*^*FL/FL*^ mice also used in (**L**) (normal water) but different representative images are shown. IHC staining (**G**) and quantification (**H**) of malondialdehyde (MDA) in *Albumin-Cre; p53*^*WT/WT*^ (WT) and *Albumin-Cre; p53*^*FL/FL*^ (FL) mice at indicated times (hours) after CCl_4_ treatment. Scale bars 20 μm. Quantification from *N* = 6 untreated (0 h) mice/group, *N* = 7 WT and N = 6 FL mice at 24 h, *N* = 7 mice/group at 48 and 72 h, and N = 3 mice/group at 168 h. Data presented as mean ± SEM and analysed using two-way ANOVA with Holm-Sidak’s multiple comparisons test and multiplicity-adjusted *p* values. **p < 0.01, ****p < 0.0001. Data from *p53*^*FL/FL*^ mice also used in (**J**) (normal water) but different representative images are shown. IHC staining (**I**) and quantification (**J**) of malondialdehyde (MDA) in *Albumin-Cre; p53*^*FL/FL*^ (FL) mice given control (water) or 30 mM N-Acetylcysteine-supplemented drinking water (NAC) for one week prior to CCl_4_ treatment. Images from indicated times (hours) after CCl_4_ treatment. Scale bars 20 μm. Quantification from *N* = 6 water and *N* = 2 NAC untreated mice (0 h), *N* = 6 water and *N* = 4 NAC mice at 24 h, *N* = 7 water and *N* = 4 NAC mice at 48-72 h. Data presented as mean ± SEM and analysed using two-way ANOVA with Holm-Sidak’s multiple comparisons test and multiplicity-adjusted p-values. **p < 0.01, ****p < 0.0001. Data from *p53*^*FL/FL*^ (normal water) mice also used in (**H**) but different representative images are shown. Staining (**K**) and quantification (**L**) of oil-red-O (ORO) in *Albumin-Cre; p53*^*FL/FL*^ (FL) mice given control (water) or 30 mM N-Acetylcysteine-supplemented drinking water (NAC) for one week prior to CCl_4_ treatment. Images from indicated times (hours) after CCl_4_ treatment. Scale bars 20μm. Quantification from *N* = 5 untreated water (0 h) and *N* = 2 untreated NAC mice, *N* = 5 water and *N* = 4 NAC mice at 24 and 48 h, *N* = 7 water and *N* = 4 NAC mice at 72 h. Data presented as mean ± SEM and analysed using two-way ANOVA with Holm-Sidak’s multiple comparisons test and multiplicity-adjusted p-values. *p < 0.05. Data from *p53*^*FL/FL*^ (normal water) mice also used in (**F**) but different representative images are shown.

Using a p53 reporter mouse, we have previously detected strong induction of the p53 pathway, albeit without clear accumulation of p53 itself, in hepatocytes of mice treated with the hepatotoxin carbon tetrachloride (CCl_4_). To explore the potential roles for p53 function during this process, we created mice harbouring liver-specific deletion of *Trp53* (*p53*) (*Albumin-Cre; p53*^*FL/FL*^ mice), confirmed that recombination of the *p53* floxed allele was highly efficient in the liver (Fig. S1A/B) and proceeded to characterise the acute response to CCl_4_-mediated liver toxicity using this model (Figs. 1A/B and S1 C–H). Importantly, mice of either *p53* genotype developed normally and were histologically indistinguishable prior to treatment (Fig. S1 C/D). Within the first 24 h following treatment with CCl_4_, *Albumin*-*Cre*; *p53*^*WT/WT*^ mice *(p53* WT) mice exhibited evident liver damage, including vacuolisation, neutrophil infiltration, and destruction of Glutamine Synthetase (GS)-positive peri-central vein hepatocytes (Figs. 1A/B and S1C–H). Damage progressed outward from the central vein over the first 48–72 hours after treatment before resolving within the remainder of the 168-h time-course (Figs. 1A/B and S1 C/D). In *Albumin-Cre; p53*^*FL/FL*^ mice, although GS-positive hepatocyte destruction and damage-associated neutrophil infiltration were similar to *p53* WT mice (Fig. S1E–H), liver damage progressed outward from the peri-central vein region more rapidly, coalescing into larger regions of injury at 24 h after CCl_4_ treatment (Figs. 1A/B and S1 C/D). Even so, the liver damage in *Albumin-Cre; p53*^*FL/FL*^ mice also resolved within 168 h.

To confirm our histological assessment of liver damage, we examined the presence of alanine and aspartate transaminase (ALT/AST) activity in blood plasma, two markers of liver damage. In agreement with the liver histology, we noted extended elevation of plasma ALT and AST activity in *Albumin-Cre; p53*^*FL/FL*^ mice (Fig. 1C/D), suggesting elevated liver damage in these mice. Even so, as observed in liver histology, plasma ALT and AST levels ultimately normalised by 168 h after treatment in mice of both genotypes, suggesting potentially significant but transient effects of p53 during CCl_4_-mediated liver regeneration. These findings are consistent with previous reports of p53 acting to limit liver damage after acetaminophen overdose [18, 19], where p53 exerted short-lived protective effects.

One of the features of damage-mediated liver regeneration is the dramatic but transient microvesicular steatosis that occurs prior to initiation of the proliferative phase of the regenerative response [28–30]. The resulting accumulation of lipids can be visualised as red puncta in oil-red O-stained liver sections. In contrast to our liver damage assessments, we observed a similar peak accumulation of oil-red-O staining at 24 h after CCl_4_ treatment in the livers of *Albumin*-*Cre*; *p53*^WT/WT^ and *Albumin-Cre; p53*^*FL/FL*^ mice (Fig. 1E/F). However, while lipid levels rapidly normalised by 48 h after treatment in *Albumin*-*Cre*; *p53*^WT/WT^ mice, elevated oil-red-O staining persisted in *Albumin-Cre; p53*^*FL/FL*^ mice for an additional 2 days (Fig. 1E/F).

The initial reductive dehalogenation of CCl_4_ generates short-lived but highly reactive intermediates that potently oxidise lipids and cause DNA damage [31, 32]. Peroxidized lipids can impede mitochondrial function, including fatty acid oxidation, and impair lipid export—potentially contributing to lipid accumulation during CCl_4_ detoxification [33–35]. Considering the established role for p53 supporting the redox response [36], we investigated whether the disrupted lipid clearance in *Albumin-Cre; p53*^*FL/FL*^ mice was a consequence of decreased ROS detoxification. Indeed, levels of malondialdehyde (MDA), a marker of lipid peroxidation, were elevated to a greater extent, and for an additional two days, in *Albumin-Cre; p53*^*FL/FL*^ mice compared with *Albumin-Cre; p53*^*WT/WT*^ mice (Fig. 1G/H).

To investigate the contribution of ROS stress to deficiencies in liver regeneration in *Albumin-Cre; p53*^*FL/FL*^ mice, we compared CCl_4_-mediated regeneration between *Albumin-Cre; p53*^*FL/FL*^ mice given normal drinking water to those provided with N-Acetylcysteine (NAC)-supplemented drinking water. NAC treatment is an established antidote to liver toxicity that results from acetaminophen overdose in humans and functions by maintaining liver glutathione levels during the detoxification process [37]. As expected, NAC treatment significantly attenuated lipid peroxidation in *Albumin-Cre; p53*^*FL/FL*^ mice (Fig. 1I/J). NAC treatment also promoted significantly more rapid clearance of lipid droplets (Fig. 1K/L), suggesting that redox management is an important feature of the p53-mediated response to hepatic CCl_4_ toxicity.

### Liver p53 engages *Cyp2a5/CYP2A6* to support redox control during CCl_4_-mediated liver regeneration

**Fig. 2:**
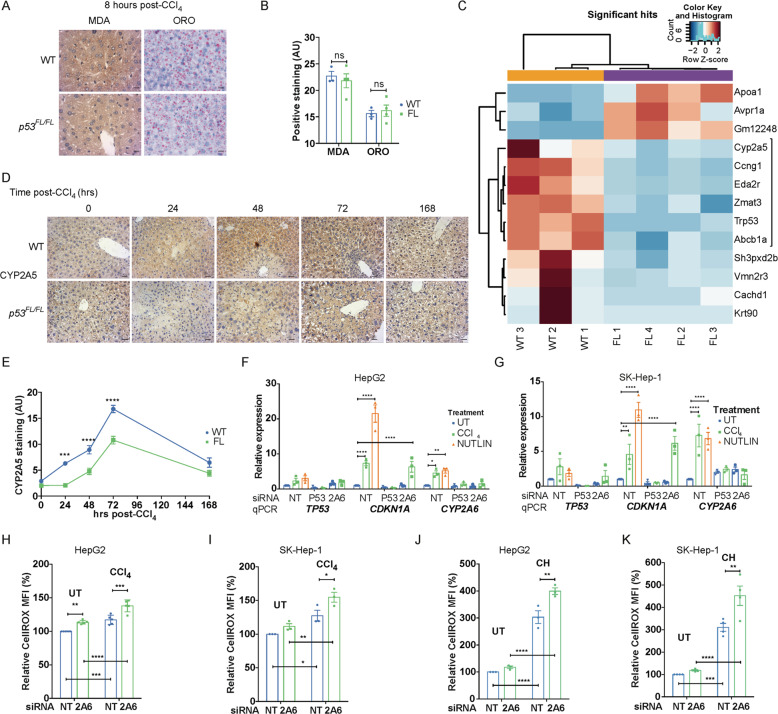
Liver p53 engages *Cyp2a5/CYP2A6* to support redox control during CCl4-mediated liver regeneration. Staining (**A**) and quantification (**B**) for malondialdehyde (MDA) and oil-red-O (ORO) in *Albumin-Cre; p53*^*WT/WT*^ (WT) and *Albumin-Cre; p53*^*FL/FL*^ (FL) mice at 8 h after CCl_4_ treatment. Scale bars 10μm. Representative of *N* = 3 WT and *N* = 4 FL mice. Data presented as mean ± SEM and analysed using two-tailed t-tests and the Sidak–Bonferroni method to account for multiplicity of tests. ns not significant. **C** Clustering analysis of significant differentially expressed genes (adjusted *p* < 0.05) from RNA-seq analysis between *Albumin-Cre; p53*^*WT/WT*^ (WT) and *Albumin-Cre; p53*^*FL/FL*^ (FL) mice at 8 h after CCl_4_ treatment. Samples from *N* = 3 WT and *N* = 4 FL mice included in analysis. Positive *Z*-score values correspond to genes enriched in livers of mice of the genotype indicated in the sample name. *Cyp2a5-*associated cluster as indicated. For further information, see materials and methods. IHC staining (**D**) and quantification (**E**) of CYP2A5 in *Albumin-Cre; p53*^*WT/WT*^ (WT) and *Albumin-Cre; p53*^*FL/FL*^ (FL) mice at indicated times (hours) after CCl_4_ treatment. Scale bars 20 μm. Quantification from *N* = 4 untreated (0 h) mice/group, *N* = 5 mice/group at 24 h, *N* = 7 mice/group at 48 and 72 h, and *N* = 3 mice/group at 168 h. Data presented as mean ± SEM and analysed using two-way ANOVA with Holm–Sidak’s multiple comparisons test and multiplicity-adjusted *p* values. ***p < 0.001, ****p < 0.0001. RT-qPCR analysis of expression of *TP53*, *CDKN1A* and *CYP2A6* relative to *ACTIN* in HepG2 (**F**) and SK-Hep-1 (**G**) cells treated with siRNA against *TP53, CYP2A6* (2A6), or non-targeting control (NT) 96 h prior to analysis and additionally treated with either DMSO control (UT), CCl_4_ (4 mM), or with Nutlin (10 μM) for 24 h prior to analysis. *N* = 3 independent samples/condition. Data presented as mean ± SEM and analysed using two-way ANOVA with Holm-Sidak’s multiple comparisons test and multiplicity-adjusted *p* values. *p < 0.05, **p < 0.01, ****p < 0.0001. Measurement of cellular ROS levels relative to baseline using the CellROX fluorescent probe in HepG2 (**H**) and SK-Hep-1 (**I**) cells treated with non-targeting control (NT) or *CYP2A6* (2A6) siRNA for 96 hours and additionally treated with either DMSO control (UT) or with CCl_4_ (4 mM) in DMSO for 24 h prior to analysis. *N* = 5 independent HepG2 and *N* = 3 SK-Hep-1 samples/condition. Data presented as median fluorescent intensity (MFI) ± SEM relative to untreated NT cells and analysed using two-way ANOVA with Holm–Sidak’s multiple comparisons test and multiplicity-adjusted *p* values. *p < 0.05, **p < 0.01, ***p < 0.001, ****p < 0.0001. Measurement of cellular ROS levels relative to baseline using the CellROX fluorescent probe in HepG2 (**J**) or SK-Hep-1 cells (**K**) treated with non-targeting control (NT) or *CYP2A6* (2A6) siRNA for 96 h and additionally treated with either DMSO control (UT) or with cumene hydroperoxide (10 μM) (CH) for 24 h prior to analysis. *N* = 3 independent samples/condition in HepG2 cells and *N* = 4/condition in SK-Hep-1 cells. Data presented as median fluorescent intensity (MFI) ± SEM relative to untreated NT cells and analysed using two-way ANOVA with Holm–Sidak’s multiple comparisons test and multiplicity-adjusted *p* values. **p < 0.01, ***p < 0.001, ****p < 0.0001.

Given the differences in redox control between *Albumin-Cre; p53*^*WT/WT*^ and *Albumin-Cre; p53*^*FL/FL*^ mice at 24 h after CCl_4_ treatment, we focused on the early response to toxicity. At 8 h after CCl_4_ treatment, we observed similar oil-red-O staining to baseline and comparable lipid peroxidation between livers taken from *Albumin-Cre; p53*^*WT/WT*^ and *Albumin-Cre; p53*^*FL/FL*^ mice (Fig. 2A/B), suggesting that bifurcation of the regenerative response had not yet occurred. Bulk RNA-seq analysis at this time point identified 13 significant differentially regulated genes between *Albumin-Cre; p53*^*WT/WT*^ and *Albumin-Cre; p53*^*FL/FL*^ livers 8 h after CCl_4_ treatment (Fig. S2A). Clustering analysis stratified these genes into three groups, one of which contained p53 itself and four established p53 targets: *Ccng1/Cyclin G1, Eda2r, Zmat3/Wig-1*, and *Abcb1a/Mdr1* [38–41] (Fig. 2C). Our attention was drawn to the remaining member of this cluster, *Cyp2a5*, encoding a cytochrome P450 enzyme that is induced by NFE2L2 (NRF2) to aid in the murine redox response during ethanol detoxification [42, 43]. *CYP2A6*, the human orthologue of *Cyp2a5* [44], has been shown to be a transcriptional target of p53 [45], suggesting potential p53-directed functions for *Cyp2a5* in the mouse as well.

Consistent with our RNA-seq data, we confirmed that expression of *Cyp2a5* was induced in *Albumin-Cre; p53*^*WT/WT*^ mice within the first 8–24 h after CCl_4_ treatment, with later induction observed in *Albumin-Cre; p53*^*FL/FL*^ mice alongside increased levels of the NRF2 target gene *Nqo1* (Fig. S2B/C). Through IHC staining, we confirmed higher levels of CYP2A5 protein in livers of *Albumin-Cre; p53*^*WT/WT*^ compared to *Albumin-Cre; p53*^*FL/FL*^ mice within 24 h after CCl_4_ treatment as well (Fig. 2D/E). CYP2A5 levels remained significantly elevated in Albumin-Cre; p53^WT/WT^ livers at 48 and 72 h after treatment, with a delayed increase evident in *Albumin-Cre; p53*^*FL/FL*^ livers at 48–72 h after CCl_4_ treatment (Fig. 2D/E).

Interestingly, although we observed potent early induction of the p53 target gene *Cdkn1a/p21* in *Albumin-Cre; p53*^*WT/WT*^ mice, this was matched by similar induction in *Albumin-Cre; p53*^*FL/FL*^ mice, a finding that we confirmed by IHC staining for p21 at 8 h after CCl_4_ treatment (Fig. S2D/E). These findings suggest that early expression of p21 after CCl_4_ treatment is p53-independent (Fig. S2D/E), in contrast to its p53-dependent induction later in the regenerative process (Fig. S2D). These results also explain why *Cdkn1a* was not differentially expressed in our RNA-seq analysis. Expression of CYP2A5, in contrast, was elevated in *Albumin-Cre; p53*^*WT/WT*^ mice but significantly lower in *Albumin-Cre; p53*^*FL/FL*^ livers at this time point, consistent with our RNA-seq data (Fig. S2F). *Bbc3*/*Puma*, a pro-apoptotic p53 target gene that has been shown to play a role in modulating liver metabolism in human HCC [46], was not differentially expressed between *Albumin-Cre; p53*^*WT/WT*^ and *Albumin-Cre; p53*^*FL/FL*^ mice during CCl_4_-mediated regeneration (Fig. S2G), reinforcing the idea that not all aspects of p53 activity are engaged during liver regeneration.

In the *Mdm2*^*Ex5/6Δ*^ mouse model, excision of *Mdm2* exons 5 and 6 (*Mdm2*^*Ex5/6Δ*^), comprising the p53-binding domain of MDM2, leads to rapid stabilisation of p53, robust expression of p53 target genes, and ultimately in p53-dependent lethality within 4–5 days [24, 47]. RNA-seq analysis of mice sampled at two days after treatment with liver-specific AAV8-TBG-Cre [48] to induce expression of *Mdm2*^*Ex5/6Δ*^, a time point before widespread liver attrition, confirmed significant induction of *Cyp2a5*. Indeed, we identified all of the genes in our CCl_4_ RNA-seq analysis, alongside classical p53 targets such as *p21* and *Puma* in this alternative model (Fig. S2H). As in the CCl_4_ liver regeneration model, we validated the induction of p21 and CYP2A5, as well as stabilisation of p53, via IHC (Fig. S2I–K). Combined, these findings confirm that activation of p53 engages CYP2A5 in the liver.

To explore the role of CYP2A5/CYP2A6 in damaged hepatocytes more fully in vitro, we turned to HepG2 and SK-Hep-1 cells, human HCC cell lines that maintain wild type p53 [46] (Fig. 2F/G). Treatment of these cells with Nutlin, a direct activator of p53 [49], induced expression of *CDKN1A/p21*, as expected, as well as *CYP2A6*. Treatment of both HepG2 and SK-Hep-1 cells with CCl_4_ also induced *p21* and *CYP2A6* expression (Fig. 2F/G). This response was abrogated following siRNA-mediated depletion of *TP53*, confirming the role of p53 in the upregulation of *p21* and *CYP2A6* expression in response to CCl_4_ in vitro (Fig. 2F/G).

Functionally, both HepG2 and SK-Hep-1 cells treated with CCl_4_ exhibited increased ROS levels, and this was exacerbated in *CYP2A6*-depleted cells (Fig. 2H/I). Treatment of HepG2 and SK-Hep-1 cells with cumene hydroperoxide (CH), a stable organic oxidising agent [50], similarly engaged *CYP2A6* and *CDKN1A/p21* (Fig. S2 L/M), and *CYP2A6*-depletion also increased cellular ROS levels after CH treatment (Fig. 2J/K)—suggesting that downstream ROS, rather than CCl_4_ directly, promotes activation of CYP2A6 to aid in ROS detoxification. Interestingly, although induction of *CYP2A6* was p53-dependent in response to CCl_4_ treatment, *CYP2A6* increased independently of p53 after CH treatment (Fig. S2L/M). Since hydroperoxides have been shown to activate NRF2 in HepG2 cells [51], this finding is consistent with an established role for NRF2-induced *Cyp2a5* supporting the redox response during ethanol detoxification in mice [42, 43]. Based on these findings, we concluded that the p53-dependent activation of CYP2A5 in response to CCl_4_ treatment in vivo contributed to the enhanced detoxification of lipid ROS in support of rapid regeneration in *Albumin-Cre*; *p53*^*WT/WT*^ mice.

### Hepatocyte p53 protects liver function and limits tumourigenesis following CCl_4_-mediated chronic regeneration

A close relationship has been described between chronic regeneration and cancer—with tumourigenesis sometimes conceptualised as ‘a wound that does not heal’ [52]. While we detected clear defects in redox control and liver function during one round of CCl_4_ treatment and regeneration in *Albumin-Cre; p53*^*FL/FL*^ mice compared to *Albumin-Cre; p53*^*WT/WT*^ mice, these differences were transient and resolved within one week (Fig. 1). In contrast to acute damage, repeated regeneration resulting from regular CCl_4_ treatment causes lasting fibrotic liver damage, leading to cirrhosis and HCC [53, 54]. This progression is exacerbated by systemic DNA damage, chronic inflammation, and ROS stress [55, 56]. With these findings in mind, we investigated the effects of lack of liver p53 on fibrosis and HCC development in the well-established CCl_4_ chronic liver regeneration model [5] (Fig. S3A).

**Fig. 3:**
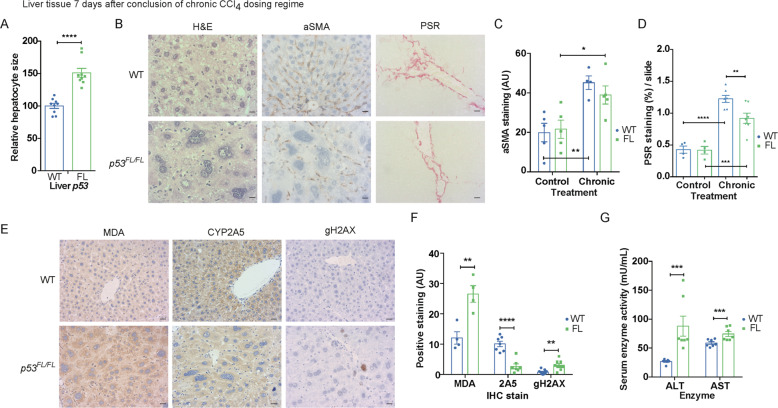
Loss of liver p53 leads to hepatic hypertrophy, chronic ROS and DNA damage, and impaired liver function after CCl4-mediated chronic regeneration. **A** Quantification of relative hepatocyte size in *Albumin-Cre; p53*^*WT/WT*^ (WT) and *Albumin-Cre; p53*^*FL/FL*^ (FL) mice at 7 days after completion of 10-week CCl_4_ chronic regeneration regime. *N* = 9 mice/genotype. Data presented as mean ± SEM and analysed using an unpaired two-tailed *t*-test with Welch’s correction. **p < 0.01. **B** H&E and IHC staining for alpha-smooth muscle actin (aSMA), and picrosirius red (PSR) staining in *Albumin-Cre; p53*^*WT/WT*^ (WT) and *Albumin-Cre; p53*^*FL/FL*^ (FL) mice at 7 days after completion of 10-week CCl_4_ chronic regeneration regime. Scale bars 10 μm. Images representative of *N* = 10 WT and *N* = 8 FL mice for H&E, *N* = 4 WT and *N* = 5 FL mice for aSMA, and *N* = 7 mice/group for PSR. **C** Quantification of IHC staining for aSMA in *Albumin-Cre; p53*^*WT/WT*^ (WT) and *Albumin-Cre; p53*^*FL/FL*^ (FL) mice from (B) at either 7 days after completion of 10-week CCl_4_ chronic regeneration regime (chronic) or in untreated age-matched mice (control). *N* = 5 control mice/genotype, *N* = 4 WT and *N* = 5 FL chronic mice. Data presented as mean ± SEM and analysed using two-way ANOVA with Holm–Sidak’s multiple comparisons test and multiplicity-adjusted *p* values. **p < 0.01. **D** Quantification of staining for picrosirius red (PSR) in *Albumin-Cre; p53*^*WT/WT*^ (WT) and *Albumin-Cre; p53*^*FL/FL*^ (FL) mice as in (**C**). *N* = 4 control mice/genotype and *N* = 7 chronic CCl_4_ mice/genotype. Data presented as mean ± SEM and analysed using two-way ANOVA with Holm–Sidak’s multiple comparisons test and multiplicity-adjusted *p* values. **p < 0.01, ***p < 0.001, ****p < 0.0001. Images (**E**) and quantification (**F**) of IHC staining MDA, CYP2A5, and gH2AX in *Albumin-Cre; p53*^*WT/WT*^ (WT) and *Albumin-Cre; p53*^*FL/FL*^ (FL) mice at 7 days after completion of 10-week CCl_4_ chronic regeneration regime as in (**C**). Scale bars 20 μm. *N* = 4 mice/group for MDA, *N* = 7 mice/group for CYP2A5, and *N* = 9 mice/group for gH2AX. Data presented as mean ± SEM and analysed using two-tailed *t*-tests and the Sidak–Bonferroni method to account for multiplicity of tests. **p < 0.01, ****p < 0.0001. **G** Plasma ALT and AST activity (mU/mL) in *Albumin-Cre; p53*^*WT/WT*^ (WT) and *Albumin-Cre; p53*^*FL/FL*^ (FL) mice at 7 days after completion of 10-week CCl_4_ chronic regeneration regime. *N* = 7 mice/group. Each data point represents the mean from technical duplicates per mouse. Data presented as mean ± SEM and analysed using two-tailed *t*-tests and the Sidak–Bonferroni method to account for multiplicity of tests. ***p < 0.001.

One week after the conclusion of the 10-week chronic regeneration regime, we observed striking generalised hepatocyte hypertrophy [57] throughout the livers of *Albumin-Cre; p53*^*FL/FL*^ mice that was absent in similarly treated *Albumin-Cre; p53*^*WT/WT*^ mice (Fig. 3A/B). In hepatic stellate cells (HSCs), p53 has been shown to limit fibrosis after chronic regeneration in the liver [5]. However, we found that hepatocyte-specific p53 loss did not lead to differences in activated HSC content, as evaluated by IHC staining for alpha-smooth muscle actin (aSMA), or to increased fibrosis as assessed by picrosirius red staining (PSR) (Fig. 3B–D). In fact, we observed a modest decrease in fibrosis in *Albumin-Cre; p53*^*FL/FL*^ mice (Fig. 3B/D), consistent with a previous report showing that hepatocyte p53 can enhance fibrosis during CCl_4_-mediated chronic regeneration in rats [27]. Although murine hepatocyte p53 does not appear to limit fibrosis after chronic regeneration either, we nevertheless detected higher levels of unresolved DNA damage, measured by IHC staining for phospho-histone H2A.X (gH2AX), increased lipid peroxidation (measured by MDA) and—as expected—decreased levels of CYP2A5 in livers from *Albumin-Cre; p53*^*FL/FL*^ mice (Fig. 3E/F). Functionally, we also found that plasma levels of ALT and AST enzyme activity were both elevated in *Albumin-Cre; p53*^*FL/FL*^ mice after chronic regeneration, consistent with compromised liver function in these mice (Fig. 3G).

**Fig. 4:**
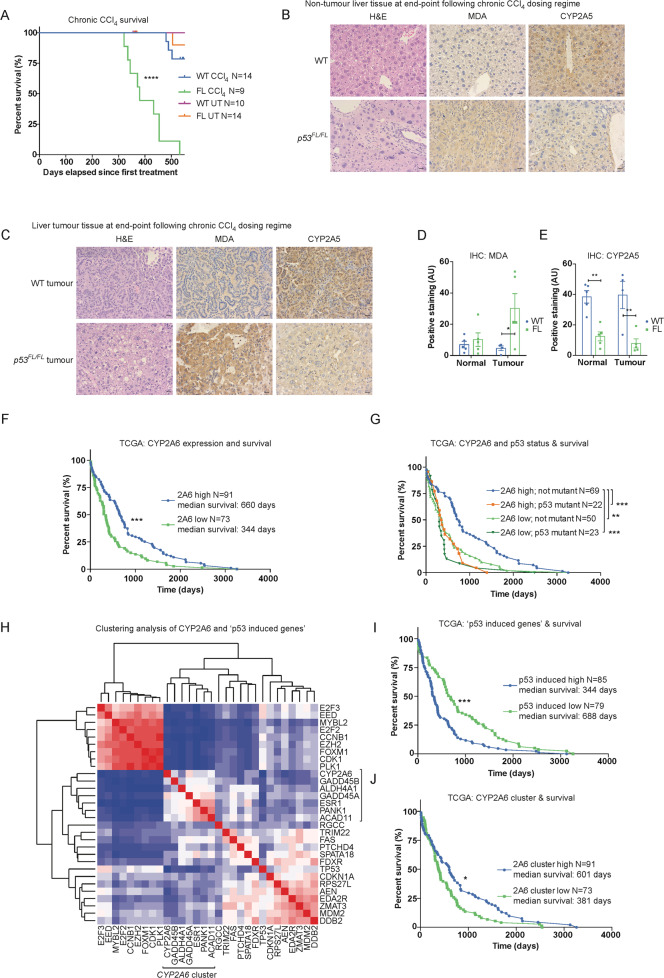
Liver p53 controls ROS, maintains expression of CYP2A5, and limits tumourigenesis following CCl4-mediated chronic regeneration. **A** Survival curve comparing *Albumin-Cre; p53*^*WT/WT*^ (WT) and *Albumin-Cre; p53*^*FL/FL*^ (FL) mice that were either untreated (UT) or administered the 10-week CCl_4_ chronic regeneration regime and aged until either reaching clinical endpoint or 550 days after initiation of treatment. Data presented as time (days) since initial CCl_4_ injection. *N* = 10 WT and *N* = 14 FL untreated mice, *N* = 14 WT and *N* = 9 FL chronic CCl_4_-treated mice. Of these, 0/10 WT and 1/14 FL UT mice and 3/14 WT and 9/9 FL CCl_4_-treated mice reached clinical endpoint within 550 days. All WT tumour mice (3/3) and the majority of FL endpoint tumour mice (5/9) exhibited focal tumour lesions along with substantial non-tumour (normal-like) tissue. These regions were sampled as ‘tumour’ and ‘non-tumour’ tissue for subsequent analyses. Within these cohorts, *N* = 4 WT and *N* = 4 FL untreated mice were examined at ~365 days and confirmed to be tumour free. Data analysed using Log-rank (Mantel–Cox) test. ****p < 0.0001. H&E and IHC staining for MDA and CYP2A5 in non-tumour liver tissue (**B**) and tumour tissue (**C**) from *Albumin-Cre; p53*^*WT/WT*^ (WT) and *Albumin-Cre; p53*^*FL/FL*^ (p53^FL/FL^) mice at clinical endpoint or 550 days post-treatment initiation after prior completion of 10-week CCl_4_ chronic regeneration regime. Scale bars 20 μm. Images representative of *N* = 5 mice/group except *N* = 4 WT tumours (2 from mice at clinical endpoint and 2 small focal tumours identified at experiment endpoint). Additional tumour images included in Supplemental Fig. 3B to illustrate staining from the diversity of tumours observed in the model. Quantification of IHC staining for MDA (**D**) and CYP2A5 (**E**) in *Albumin-Cre; p53*^*WT/WT*^ (WT) and *Albumin-Cre; p53*^*FL/FL*^ (FL) mice from (**B/C**). Data presented as mean ± SEM and analysed using two-way ANOVA with Holm–Sidak’s multiple comparisons test and multiplicity-adjusted *p* values. *p < 0.05, **p < 0.01. **F** Survival curve comparing HCC patients in the TCGA-LIHC dataset with high vs. low expression of *CYP2A6* (2A6) on the basis of median survival. *N* = 91 *CYP2A6* high and *N* = 73 *CYP2A6* low patients. Data analysed using the Log-rank (Mantel–Cox) test. ***p < 0.001. **G** Survival curve comparing HCC patients in the TCGA-LIHC dataset on the basis of *TP53* status (loss or mutation of p53 (mutant) vs. WT *TP53* (not mutant)) and further breakdown of high vs. low expression of *CYP2A6* (2A6) on the basis of median survival. *N* = 69 2A6 high, not mutant, *N* = 22 2A6 high, *TP53* mutant, *N* = 50 2A6 low, not mutant, and *N* = 23 2A6 low, *TP53* mutant. Data analysed using the Log-rank (Mantel-Cox) test. **p < 0.01, ***p < 0.001. **H** Clustering analysis of expression of *CYP2A6* related to the previously identified 20 gene ‘p53-Induced Gene Target Expression Signature’ [14] using the TCGA-LIHC dataset. *CYP2A6*-associated cluster as indicated. For further information, see materials and methods. **I** Survival curve comparing HCC patients in the TCGA-LIHC dataset on the basis of high vs. low expression of the ‘p53-Induced Gene Target Expression Signature’ [14]. *N* = 85/79 patients in the high and low groups. This analysis is independent of patient p53 status and includes all data in the TCGA-LIHC dataset. Data analysed using the Log-rank (Mantel–Cox) test. ***p < 0.001. **J** Survival curve comparing HCC patients in the TCGA-LIHC on the basis of high vs. low expression of the subset of genes within the ‘p53-Induced Gene Target Expression signature’ that clustered with *CYP2A6* expression in (**H**). *N* = 91/73 patients in the high and low groups. Data analysed using the Log-rank (Mantel–Cox) test. *p < 0.05.

In wild-type mice, it can take up to 2 years for HCC to arise from chronic CCl_4_ treatment [58]. Consistent with this, few *Albumin-Cre; p53*^*WT/WT*^ mice (3/14) reached clinical endpoint within 550 days after initial CCl_4_ treatment in our experimental cohorts (Fig. 4A). In contrast, *Albumin-Cre; p53*^*FL/FL*^ mice exhibited accelerated and highly penetrant development of liver tumours in this timeframe (9/9 mice, median survival 380 days after first CCl_4_ treatment) (Fig. 4A). Most mice of both genotypes (2/3 *Albumin-Cre; p53*^*WT/WT*^ mice and 5/9 *Albumin-Cre; p53*^*FL/FL*^ mice) reaching clinical endpoint exhibited large tumour lesions but also retained non-tumour (normal-like) tissue (Fig. S3B). These regions were sampled as ‘tumour’ and ‘non-tumour’ tissue for subsequent analyses. At the experiment endpoint (550 days after initial treatment), most CCl_4_-treated *Albumin-Cre; p53*^*WT/WT*^ mice (11/14), all untreated *Albumin-Cre; p53*^*WT/WT*^ mice (10/10), and most untreated *Albumin-Cre; p53*^*FL/FL*^ mice (13/14) remained alive.

Non-tumour liver tissue from *Albumin-Cre; p53*^*FL/FL*^ mice retained the molecular features observed at the conclusion of the CCl_4_ treatment regime, including prominent hypertrophy in remaining normal hepatocytes (Fig. 4B) and significantly reduced CYP2A5 expression (Fig. 4B, E). Analysis of tumours from *Albumin*-*Cre*; *p53*^*WT/WT*^ mice (2 from mice at clinical endpoint and 2 small focal tumours identified at experiment endpoint) revealed that expression of CYP2A5 was elevated and MDA staining was low compared to tumours arising in *Albumin-Cre; p53*^*FL/FL*^ mice (Fig. 4C–E and Fig. S3C). These results confirmed that the disparate features of regeneration in *Albumin-Cre; p53*^*FL/FL*^ mice—namely elevated ROS, persistent liver damage, and decreased CYP2A5 expression—continued during tumourigenesis.

The p53-mediated expression of *BBC3/PUMA* has been shown to promote a pro-cancer metabolic switch in human HCC, correlating with poor prognosis in patients [46]. However, we did not observe differential expression of murine *Bbc3* in endpoint HCC tumours arising in *Albumin-Cre; p53*^*WT/WT*^ mice after chronic CCl_4_ treatment compared with those in *Albumin-Cre; p53*^*FL/FL*^ mice (Fig. S3D). Expression of *Cdkn1a/p21*, in contrast, was elevated in the tumours of *Albumin-Cre; p53*^*WT/WT*^ mice (Fig. S3D). These findings suggest that in murine HCC arising from chronic CCl_4_ treatment the p53-PUMA mediated metabolic switch [46] is not a defining feature of *p53* WT HCC.

### *CYP2A6* expression is prognostically favourable and clusters with a subset of p53-induced genes that correlate with increased survival in human HCC

Given the strength of the association between increased survival, retention of p53, and expression of CYP2A5 in the murine CCl_4_ chronic regeneration model, we examined whether high expression of *CYP2A6* correlated with increased survival in human HCC patients. Utilising the HCC dataset available through the cancer-genome atlas (TCGA-LIHC dataset [59]), we confirmed that high expression of *CYP2A6* was associated with significantly greater median survival in HCC (Fig. 4F). In addition, by stratifying the TCGA dataset between patients with disrupted *TP53* (mutation or loss) and those with WT *TP53*, we further determined that high expression of *CYP2A6* and retention of WT *TP53* coincided with increased survival compared with all other combinations of tumours harbouring low expression of *CYP2A6* and/or loss of WT *TP53* (Fig. 4G).

Previous work utilising TCGA data has focused on the genomic determinants of human HCC [14]. In these analyses, the authors’ identified a ‘p53-induced gene target expression signature’ as part of an aim to improve the clustering of HCC based on molecular and biological attributes [14]. Using the TCGA-LIHC dataset, we examined the relationship between these 20 identified p53-induced genes and *CYP2A6* expression and found that *CYP2A6* expression clustered with a subset of the p53-induced genes including *GADD45B*, *ALDH4A1, GADD45A*, *ESR1*, *PANK1*, and *ACAD11* (Fig. 4H). Interestingly, although high expression of the full p53-induced gene target expression signature correlated with significantly reduced median survival in the TCGA-LIHC dataset (Fig. 4I), we found that high expression of the *CYP2A6*-associated gene cluster instead correlated with improved median survival (Fig. 4J). Taken together, these observations are consistent with a role for p53 and CYP2A6 in limiting liver cancer.

## Discussion

Tissue regeneration recapitulates many features of tumourigenesis, including potent activation of proliferative signalling pathways, changes to cellular metabolism, and rapid cell growth [60]. With this overlap in mind, we have examined the function of the canonical tumour suppressor protein p53 during regeneration. Using the liver as a model system, we have interrogated non-tumour roles for p53 activity during both the acute and chronic responses to the liver toxin and carcinogen CCl_4._ We identified p53-mediated redox control and induction of CYP2A5/CYP2A6 as important features of the hepatic p53 response both in vivo and in human liver cancer cell lines in vitro.

Our findings suggest that a p53 programme is active during the priming phase of acute CCl_4_-mediated regeneration that includes induction of *Cyp2a5*, a cytochrome P450 enzyme that can be induced to control ROS in the liver [42, 43, 61]. In *TP53* WT human HCC cell lines in vitro, we show that *CYP2A6*, the human orthologue to murine *Cyp2a5*, is similarly engaged in a p53-dependent manner in response to CCl_4_ or Nutlin treatment, as well as in response to treatment with a ROS-inducing agent, cumene hydroperoxide. In treated cells, the loss of *CYP2A6* exacerbates redox stress, confirming the role of *CYP2A6* in supporting ROS control. Consistent with this observation, we show that p53 acts to limit the propagation of CCl_4_-mediated damage by controlling resulting ROS. These ROS-control activities, likely alongside additional functions of p53, promote rapid regeneration and restoration of normal liver function that is delayed in the absence of liver p53.

Our results are somewhat at odds with a previous report where *Cyp2a5* was not shown to play a significant role in the hepatic response to CCl_4_ toxicity [62]. However, this study examined CYP2A5 activity only at 24 h after administration of a significantly smaller dose of CCl_4_ to initiate acute regeneration—leading to markedly less liver damage than observed in our model. This discrepancy raises the interesting possibility that p53 activation may require a threshold of liver damage, ROS, or other stimuli to be sufficiently engaged. Given that DNA damage occurs during CCl_4_ toxicity, the severity of induced DNA damage may play a role, and future work examining this prospect could clarify the activating signals that direct p53 during liver regeneration.

During the repeated damage of CCl_4_-mediated chronic liver regeneration, we found that the paradigms identified in acute regeneration persist. The presence of hepatic p53 does not limit, but rather leads to a slight increase in fibrosis in our model. Nevertheless, as in acute regeneration, p53 continues to engage CYP2A5, restrict lipid peroxidation, and maintain liver architecture and function. These protective actions are blunted in livers that lack p53, leading to pervasive hepatocyte hypertrophy, chronically increased ROS, unresolved DNA damage, and ultimately to mortality from liver cancer. Thus, in our system, increased fibrosis is not required for increased tumourigenesis. Further work is warranted to more carefully examine the relationship between fibrosis, p53 signalling, and liver tumourigenesis.

Our findings generalise to human HCC, where high expression of *CYP2A6* correlates with increased median survival, as well as increased survival in the subset of patients that retain WT *TP53* and maintain high expression of *CYP2A6*. These results suggest that increased *CYP2A6* expression is an important component of p53’s tumour-suppressive function. Even so, our results also suggest that elevated *CYP2A6* alone is not sufficient to substitute for p53 activity in limiting liver tumorigenesis, consistent with the diverse repertoire of p53 tumour-suppressive activities in the cell. We have further distinguished a group of six genes previously reported as part of a p53-induced gene signature in human HCC [14] whose expression clusters with *CYP2A6* and together account for improved median survival—in contrast with the poor prognosis associated with high expression of the entire gene set.

Focusing on this point, we were surprised that high expression of the whole p53 gene signature significantly reduced median patient survival. However, it has been shown that p53 can help to protect cancer cells from nutrient starvation [63, 64], reduce cell death from ferroptosis [65, 66], and enhance redox control to limit ROS [67, 68]. In addition, common tumour-derived p53 mutants have been found to retain aspects of WT p53 function that promote adaptation to metabolic stress [69, 70]. With these findings in mind, and considering that ‘pro-tumourigenic’ p53 is an established paradigm in skin carcinogenesis [71–73], it is conceivable that aspects of p53 function can also enhance tumourigenesis in the liver. Future work investigating this possibility is warranted.

In humans, expression of *CYP2A6* and of various *CYP2A6* polymorphisms have been linked to higher rates pancreatic and colorectal cancer but to mostly reduced rates of lung and oesophageal cancer [74–78]. These findings suggest tissue, and potentially carcinogen-specific, functions for *CYP2A6* in limiting or promoting tumourigenesis. In the liver, our findings suggest that expression of *CYP2A6* is beneficial. One method to infer CYP2A6 activity non-invasively is through the analysis of CYP2A6-derived urinary metabolites of caffeine [74, 75]. Increased consumption of coffee reduces the risk of developing HCC [79]. As such, it would be interesting to examine whether caffeine consumption promotes *CYP2A6* expression. If so, this pathway could account for some of the protective features of coffee consumption against HCC. Future work examining this relationship, as well as whether CYP2A6 activity in HCC patients has prognostic or stratification value, is warranted.

Taken together, our results underscore the importance of p53 for maintaining liver function following damage. Interestingly, in contrast to previous models showing that the tumour suppressor function of p53 is a reflection of its ability to drive the elimination of damaged cells [80], our work shows that the repair and survival activities of p53 can also suppress the development of HCC.

## Materials and methods

### Mice

Procedures involving mice were performed under Home Office licence numbers 70/8645, PP6345023, and 70/8891. Experiments were conducted in accordance with the Animals (Scientific Procedures) Act 1986 and the EU Directive 2010 and sanctioned by Local Ethical Review Process (University of Glasgow). Mice were housed on a 12-h light/12-h dark cycle and provided with normal chow diet and water *ad libitum*. Mice were genotyped by Transnetyx (Cordova, TN).

*p53*^FL/FL^ (*Trp53*^tm1Brn^), *Albumin*-*Cre* (*Speer6-ps1*^Tg(Alb-cre)21Mgn^), and *Mdm2*^*Ex5/6Δ*^ *(Mdm2*^*tm2.1Glo*^*)* mice were described previously [47, 81, 82].

For acute CCl_4_-mediated liver regeneration, mice were treated as previously described [53, 83]. In brief, young male *Albumin*-*Cre*; *p53*^*WT/WT*^ and *Albumin-Cre; p53*^*FL/FL*^ mice (approx. 70 days old) were given CCl_4_ (1 mL/kg from stock solution of 20% CCl_4_ v/v in corn oil) (Sigma cat# 289116 and C8267) via a single intraperitoneal (IP) injection administered in the morning. Mice receiving NAC-supplemented drinking water were provided with 30 mM NAC (Sigma cat# A7250) in water ad libitum for 72 h prior to treatment with CCl_4_ and throughout the recovery period after treatment.

For chronic CCl_4_-mediated liver regeneration, young male *Albumin*-*Cre*; *p53*^*WT/WT*^ and *Albumin-Cre; p53*^*FL/FL*^ mice (approx. 70 days old) were treated weekly with IP injections of CCl_4_ (1 mL/kg from stock solution of 20% CCl_4_ v/v in corn oil) for 10 consecutive weeks. Separate cohorts of mice were either sampled 7 days after the final injection or monitored until reaching clinical endpoint (or 550 days after the first injection) and sampled at this time.

Cohorts were composed of fully backcrossed C57BL/6J (N10) *Albumin*-Cre; *p53*^*WT/WT*^ and *Albumin-Cre; p53*^*FL/FL*^ male mice. Some mice from both genotypes also contained the *Rosa26*^*LSL-tdRFP*^ (Gt(ROSA)26Sor^tm1Hjf^) reporter allele [84], which did not affect the response to CCl_4_ treatment. The experimental unit in all of our analyses was the individual mouse. No statistical test was performed to predetermine sample size. Initial pilot studies suggested a strong effect of liver *p53* status on the response to CCl_4_ treatment 24–72 h after administration, and subsequent experiments were performed using sample sizes based on standard protocols in the field. No animals were excluded from analysis. Within each experiment, mice were age and littermate matched as much as possible, and all treated at the same time. Downstream analyses were performed on a random order of samples blinded to the genotype and treatment regime until the summation of results.

For *Mdm2*^*Ex5/6Δ*^ RNA-seq experiments, mice homozygous for the *Mdm2*^*tm2.1Glo*^ allele were bred on a mixed background. 8–12 week old male mice were injected with either AAV8.TBG.PI.Cre.rBG (Addgene, 107787-AAV8) or AAV8.TBG.PI.Null.bGH (Addgene, 105536-AAV8) at a dose of 2 × 10^11^ genetic copies/mouse, as described previously [23]. Male mice of the same age and genotype, but without AAV injection, served as baseline controls (Untreated/uninduced controls). All mice were euthanized at 48 h post-AAV injection via CO_2_ inhalation.

### Recombination PCR

For recombination PCR, liver DNA from *Albumin*-*Cre*; *p53*^*WT/WT*^ mice (WT) and liver and kidney DNA from *Albumin*-*Cre*; *p53*^*FL/FL*^ (*p53*^*FL/FL*^) adult mice were isolated as previously described [85]. DNA was amplified using KOD Hot Start Master Mix (Merck Millipore cat# 71842) according to standard protocols. PCR primers were previously described [86].

### Liver function assays (ALT/AST)

ALT and AST activity were determined in EDTA-treated plasma using the Alanine Transaminase Activity Assay Kit (ab105134) and the Aspartate Aminotransferase Activity Assay Kit (ab138878) from Abcam. Both assays were performed following the manufacturer’s recommendations. Samples were run together, analysed in duplicate wells per mouse sample, and the mean value of these technical replicates was used for subsequent analysis.

### Immunohistochemistry (IHC) and special staining

Staining for oil-red-O was performed on 10 μm frozen sections that were first fixed for 5 min in 10% neutral buffered formalin (Solmedia), rinsed in tap water, and then briefly rinsed in 60% isopropanol (Fisher Chemicals). Slides were stained in freshly prepared and filtered oil-red-O staining solution (0.5% w/v oil-red-o (Merck Life Science, UK) in isopropanol (Fisher Chemicals)) for 15 min with agitation. Slides were subsequently blotted, rinsed with 60% isopropanol and then water, before application of Mayers Haematoxylin (Sigma Aldrich) to stain the nuclei. Stained slides were first sealed using Aqueous mountant (Dako) and left overnight before being coverslipped using DPX mountant (CellPath, UK).

Staining for PSR was performed on 4 μm formalin-fixed paraffin-embedded sections that were de-waxed and rehydrated through xylene and a graded ethanol series. Rehydrated slides were stained for 2 h in PSR staining solution (equal volumes of 0.1% Direct red 80 (Sigma Aldrich) and 0.1% Fast green (Raymond A Lamb) (both in distilled water) combined in a 1:9 dilution with aqueous Picric acid solution (VWR)), rinsed in tap water, and dehydrated through a graded ethanol series and xylene before being coverslipped using DPX mountant (CellPath, UK).

Manual and automated IHC staining were performed as previously described [69, 85] with the reagents and staining platform used for each antibody as noted in the accompanying reagent and antibody information tables (Supplementary Tables 1 and 2).

### Analysis of IHC images

The analysis of IHC staining in CCl_4_ experiments, and for CYP2A5 IHC staining in *Mdm2*^*Ex5/6Δ*^ mice, was performed as previously described [69, 85].

For the analysis of IHC staining for p53 and p21 in *Mdm2*^*Ex5/6Δ*^ mice, a Leica Aperio AT2 slide scanner (Leica Microsystems, UK) was used to scan stained sections at 20× magnification. Histological scoring was performed using HALO image analysis software (V3.1.1076.363, Indica Labs).

### Quantification of liver damage

A minimum of five random non-overlapping 4× magnification fields were taken from each H&E stained slide using an Olympus BX51 microscope with Zen Blue software (Zeiss). From these images, damage was manually traced and the total damaged area per slide was calculated using imageJ software.

### Cell culture

HepG2 (HB-8065) and SK-Hep-1 (HTB-52) cells were obtained from ATCC but were not authenticated. Mycoplasma testing was performed when cells were thawed and semi-regularly thereafter using the MycoAlert Mycoplasma Detection Kit (Lonza LT07-318). Independent experiments were performed on cells treated with siRNA and compounds from separate passages of each cell line. Stock flasks were maintained in DMEM glucose, glutamine, and phenol red-free medium (Gibco, A1443001) supplemented with 4 mM glucose (Sigma cat# 49163), 1 mM pyruvate (Gibco cat# 11360088), 1 mM l-Glutamine (Gibco cat# 25030032), penicillin/streptomycin (Gibco cat# 15070063), Gentamycin (Gibco cat# 15750037), and 10% FBS (Gibco cat# 10091148). Cells were cultured at 37 °C in a humidified atmosphere of 5% CO_2_.

Cells were treated with 10 μM Nutlin-3a (Nutlin) (Sigma cat# SML0580) dissolved in DMSO, 4 mM CCl_4_ (Sigma cat# 289116) dissolved in DMSO, 10 μM cumene hydroperoxide (Thermo Fisher Scientific cat# C10445) dissolved in DMSO, or DMSO as vehicle control. For in vitro experiments, CCl_4_ was prepared by first combining an 80/20 (v/v) mixture of CCl_4_ and DMSO with media to make a 100× stock. The stock was then sonicated for 5 min to disperse the CCl_4_ mixture and the resulting solution was added to cells.

### Transfection with siRNA

Studies utilising siRNA knockdown were performed as previously described [69], with siGENOME SMARTpool siRNA constructs (Horizon) used for the non-targeting siRNA control pool (D-001206-13-05) and to target *P53* (M-003329-03-0005) and *CYP2A6* (M-008781-02-0005). Constructs were used to transfect cell lines at 20 nM concentration using the Lullaby siRNA transfection reagent and the manufacturer’s recommended reverse transfection procedure (OZ Biosciences).

### Flow cytometry

HepG2 and SK-Hep-1 cells were analysed for cellular ROS levels as previously described [69]. Data were analysed using FlowJo X 10.0.7r2 (FlowJo, LLC) and median fluorescence intensity values were obtained and compared across samples.

### RNA-seq

Liver samples were isolated and preserved in Allprotect tissue reagent (Qiagen cat# 76405) (CCl_4_ samples) or snap frozen on dry ice and stored at −80 °C until RNA extraction (*Mdm2*^*Ex5/6Δ*^ samples). To isolate RNA, tissue was homogenised using a Precellys tissue homogeniser (Bertin Instruments) and RNA was extracted using the RNeasy Plus Universal mini kit (Qiagen cat# 73404) (CCl_4_ samples) or the RNeasy mini kit (Qiagen cat# 74104) (*Mdm2*^*Ex5/6Δ*^ samples), all according to the manufacturers’ recommendations. The quality of the purified RNA was tested on an Agilent 2200 Tapestation using RNA screentape (Agilent). Libraries for cluster generation and DNA sequencing were prepared as previously described using an Illumina TruSeq Stranded mRNA LT Kit (CCl4 samples) or an Illumina TruSeq Stranded mRNA HT Kit (*Mdm2*^*Ex5/6Δ*^ samples) [87]. The quality and quantity of the DNA libraries was assessed on an Agilent 2200 Tapestation (D1000 screentape) and Qubit (Thermo Fisher Scientific), respectively. The libraries were run on the Illumina Next Seq 500 using the High Output 75 cycles kit (2 × 36 cycles, paired-end reads, single index for CCl_4_ samples and 2 × 36 cycles, paired-end reads, dual index for *Mdm2*^*Ex5/6Δ*^ samples).

### Analyses of RNA-seq expression data

For CCl_4_ RNA-seq, Fastq files were generated from the sequencer output using Illumina’s bcl2fastq (version 2.15.0.4) and quality checks on the raw data were done using FastQC (version 0.10.1) [88] and FastQ Screen (version 0.4.2) [89]. Alignment of the RNA-Seq paired-end reads was to the GRCh38.75 [90] version of the mouse genome and annotation using Tophat (version 2.0.13) with Bowtie (version 2.2.6.0) [91]. Expression levels were determined and statistically analysed by a workflow combining HTSeq (version 2.2.4.0) [92], the R environment (version 3.4.2) [93], and packages from the Bioconductor data analysis suite [94]. Differential gene expression analysis was based on the negative binomial distribution using the DESeq2 package [95]. “Heatmap.2” function of gplots package [96] was used for hierarchical clustering of significant hits.

For *Mdm2*^*Ex5/6Δ*^ RNA-seq, quality checks and trimming on the raw RNA-Seq data files were done using FastQC (version 0.11.7) [88], FastP [97] and FastQ Screen (version 0.12.0) [89]. RNA-Seq paired-end reads were aligned to the GRCh38.92 [90] version of the mouse genome and annotated using HiSat2 version 2.1.0 [98]. Expression levels were determined and statistically analysed by a combination of HTSeq version 0.9.1 [92] and the R environment version 3.4 [93], utilising packages from the Bioconductor data analysis suite [94] and differential gene expression analysis based on the negative binomial distribution using the DESeq2 package version 1.18.1 [95].

### TCGA analysis

Survival, mutation and expression data were obtained via cBioPortal [99, 100]. The results here are in whole or part based upon data generated by the TCGA Research Network (http://cancergenome.nih.gov/), using the TCGA-LIHC dataset [59].

The optimal cut-off point for high or low expression of *CYP2A6* in survival analyses was determined using the “surv_cutpoint” function of survminer package in R (0.4.8) [93, 101]. Overall survival data from patients for each expression group was plotted and analysed using inbuilt tools as indicated in Prism 7 (Graph Pad). Correlations between *CYP2A6* expression and the ‘P53-induced gene target expression signature’ [14] were assessed using the “cor” function from base R [93]. Then, the resulting heatmap was plotted using the function “corrplot” from the corrplot package (Version 0.84) to plot heatmaps [102].

### Quantitative RT-PCR

For qPCR analysis of mouse tissue, liver samples were isolated and preserved in Allprotect tissue reagent (Qiagen cat# 76405). RNA was extracted as previously described [85]. cDNA was synthesised using the high capacity RNA-to-cDNA kit (Thermo Fisher Scientific cat# 4387406) and qPCR reactions were performed on a QuantStudio 5 real-time PCR system (Thermo Fisher Scientific) using Taqman FAST advanced master mix and Taqman gene expression assays (all Thermo Scientific) according to the manufacturer’s recommendations and using the assays listed in Supplemental Table 3. Gene expression was quantified relative to the housekeeping gene *Beta-glucuronidase* according to the comparative ΔΔCt method.

For qPCR analysis of human cell lines, RNA was extracted from HepG2 and SK-Hep-1 cells using the RNeasy mini kit (Qiagen cat# 74104) according to the manufacturer’s recommendations, omitting the optional additional DNase treatment step. cDNA synthesis and qPCR reactions were performed as for mouse tissue samples described above. Gene expression in human cell lines was quantified relative to the housekeeping gene *ACTIN* according to the comparative ΔΔCt method.

### Data plotting and statistical analysis

Data were plotted using Prism 7 (Graph Pad). The statistical analysis for each experiment was performed using the test indicated in the relevant figure legend and multiplicity-adjusted *p* values using the built-in analysis tools of Prism 7. Statistical tests were chosen based on the nature of the comparison being made and the corresponding standard tests utilised in the field. Underlying assumptions for these tests, including sample independence, variance equality, and normality were assumed to be met although not explicitly examined. Figures were prepared using Illustrator (Adobe). Unless otherwise indicated, data are represented as mean ± standard error of the mean (SEM) for error bars. Asterisks denote *p* value as follows: *p < 0.05, **p < 0.01, ***p < 0.001, ****p < 0.0001.

## Supplementary information

Supplemental Material

## Data Availability

The data that support the findings of this study are available from the corresponding authors upon reasonable request. RNA-seq data discussed in this paper have been deposited in NCBI’s Gene Expression Omnibus and are accessible through GEO Series accession numbers GSE183053 and GSE183082.
