# Supplementary material for: p53-mediated redox control promotes liver regeneration and maintains liver function in response to CCl4
Source: Cell Death Differ. 2021 Oct 9;29(3):514–26. doi: 10.1038/s41418-021-00871-3 (PMC8901761; doi:10.1038/s41418-021-00871-3)
Supplement: Supplementary file 1 — Supplemental Material [file 41418_2021_871_MOESM1_ESM.pdf]

Supplemental Materials List

Supplemental Figures (S1-S3)

Supplemental Tables

Supplemental Table 1: IHC and staining reagents

Supplemental Table 2: Primary antibodies used in IHC

Supplemental Table 3: Quantitative RT-PCR primers

Humpton Supplemental Figure 1

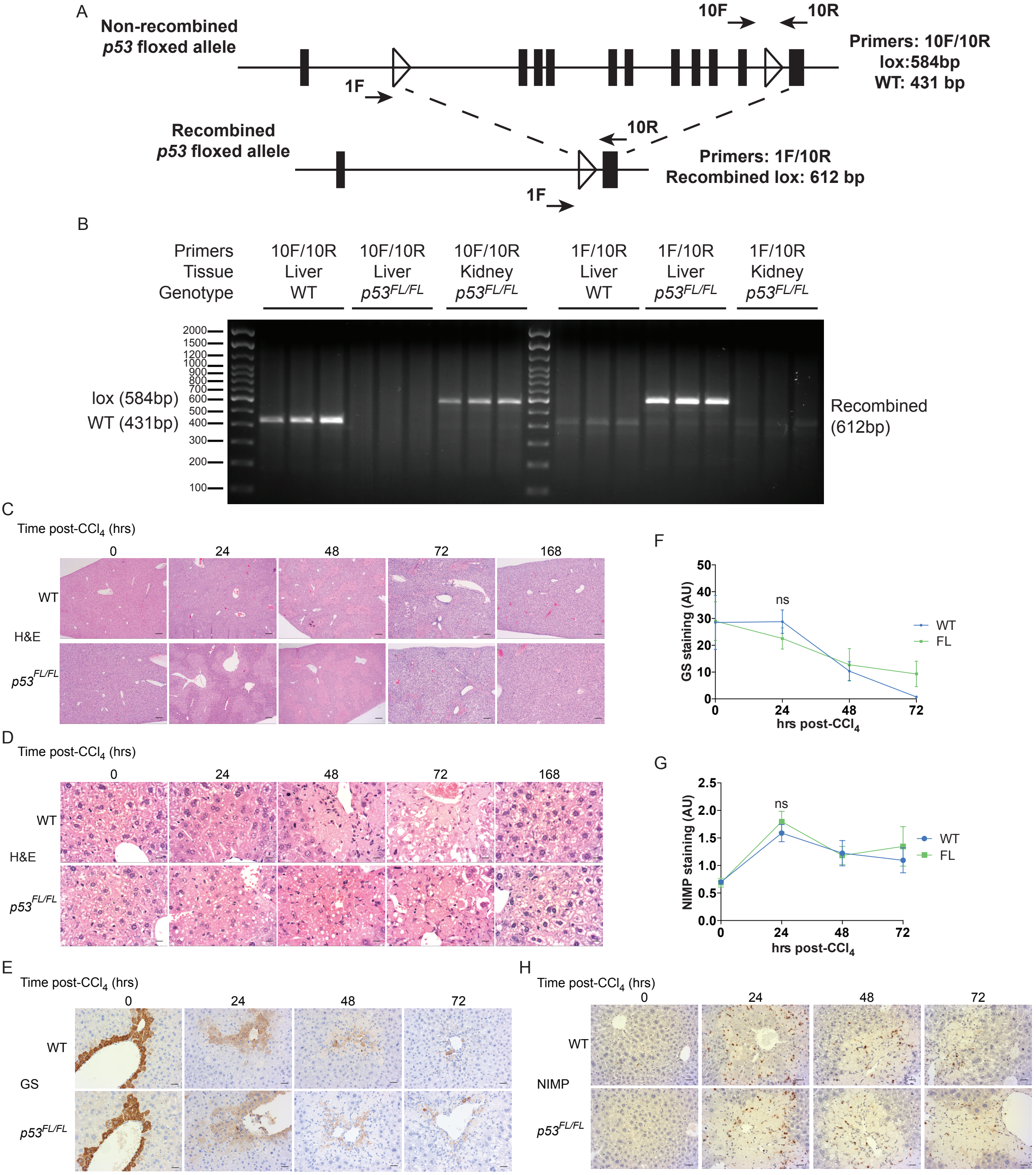

A. Schematic of *p53 floxed* allele including *loxP* sites (open triangles) and primers used to detect the non-recombined and recombined alleles (arrows).

B. PCR to assess recombination of *p53 floxed* allele in the liver of *Albumin*-Cre; $p53^{WT/WT}$ mice (WT) and the liver and kidney of *Albumin*-Cre; $p53^{FL/FL}$ ($p53^{FL/FL}$) adult mice. Expected bands for WT (431 bp) and non-recombined p53 floxed allele (584 bp) using the 10F and 10R primers, and recombined (612 bp) p53 floxed allele using the 1F and 10R primers as shown. PCR reaction run for 35 cycles for all samples. N= 3 mice/group (liver and kidney samples are from the same $p53^{FL/FL}$ mice).

C/D. Representative H&E images of livers from *Albumin*-Cre; $p53^{WT/WT}$ mice (WT) and *Albumin*-Cre; $p53^{FL/FL}$ ($p53^{FL/FL}$) mice at indicated times (hours) following CCl_4 treatment at lower (C) and higher (D) magnification. Images reproduced in Figure 1 A with annotation of damaged areas. Scale bars 100µm in (C) and 10µm in (D).

E/F. IHC staining (E) and quantification (F) for Glutamine Synthetase (GS) in *Albumin*-Cre; $p53^{WT/WT}$ mice (WT) and *Albumin*-Cre; $p53^{FL/FL}$ ($p53^{FL/FL}$) mice at indicated times (hours) following CCl_4 treatment. Images focused on central vein regions. Scale bars 20µ m. N=4 mice/group at 0 hrs and 24 hrs, N=5 mice/group at 48 and 72 hrs. Data presented as mean +/- SEM and analysed using two-way ANOVA with Holm-Sidak's multiple comparisons test and multiplicity-adjusted p-values. ns: not significant.

G/H. IHC staining (G) and quantification (H) for neutrophils using NIMP antibody in *Albumin*-Cre; $p53^{WT/WT}$ mice (WT) and *Albumin*-Cre; $p53^{FL/FL}$ ($p53^{FL/FL}$) mice at indicated times (hours) following CCl_4 treatment. Images focused on central vein regions. Scale bars 20µm. N=3 mice/group. Data presented as mean +/- SEM and analysed using two-way ANOVA with Holm-Sidak's multiple comparisons test and multiplicity-adjusted p-values. ns: not significant.

Humpton Supplemental Figure 2

A RNA-seq WT vs $p53^{FL/FL}$ @ 8 hours post-CCl_4

Gene Name	Gene Symbol	FC (FL vs WT)	p-adjusted
ectodysplasin A2 receptor	Eda2r	-3.95	6.52E-17
transformation related protein 53	Trp53	-2.44	2.71E-10
zinc finger matrin type 3	Zmat3	-0.71	5.57E-06
keratin 90	Krt90	-19.15	5.79E-05
cyclin G1	Ccng1	-1.05	7.46E-05
ATP-binding cassette, sub-family B (MDR/TAP), member 1A	Abcb1a	-1.17	9.25E-04
arginine vasopressin receptor 1A	Avpr1a	1.27	1.16E-03
SH3 and PX domains 2B	Sh3pxd2b	-0.89	2.84E-03
vomeronasal 2, receptor 3	Vmn2r3	-2.00	1.75E-02
predicted gene 12248	Gm12248	5.55	3.79E-02
apolipoprotein A-I	Apoa1	0.66	4.36E-02
cache domain containing 1	Cachd1	-2.03	4.36E-02
cytochrome P450, family 2, subfamily a, polypeptide 5	Cyp2a5	-1.84	4.63E-02

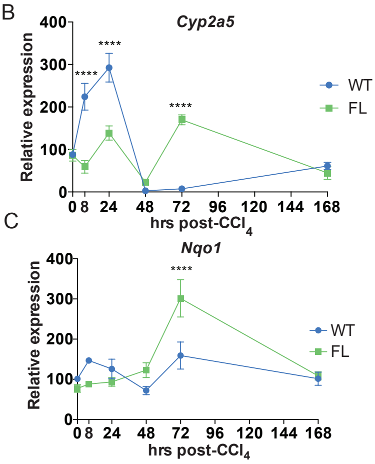

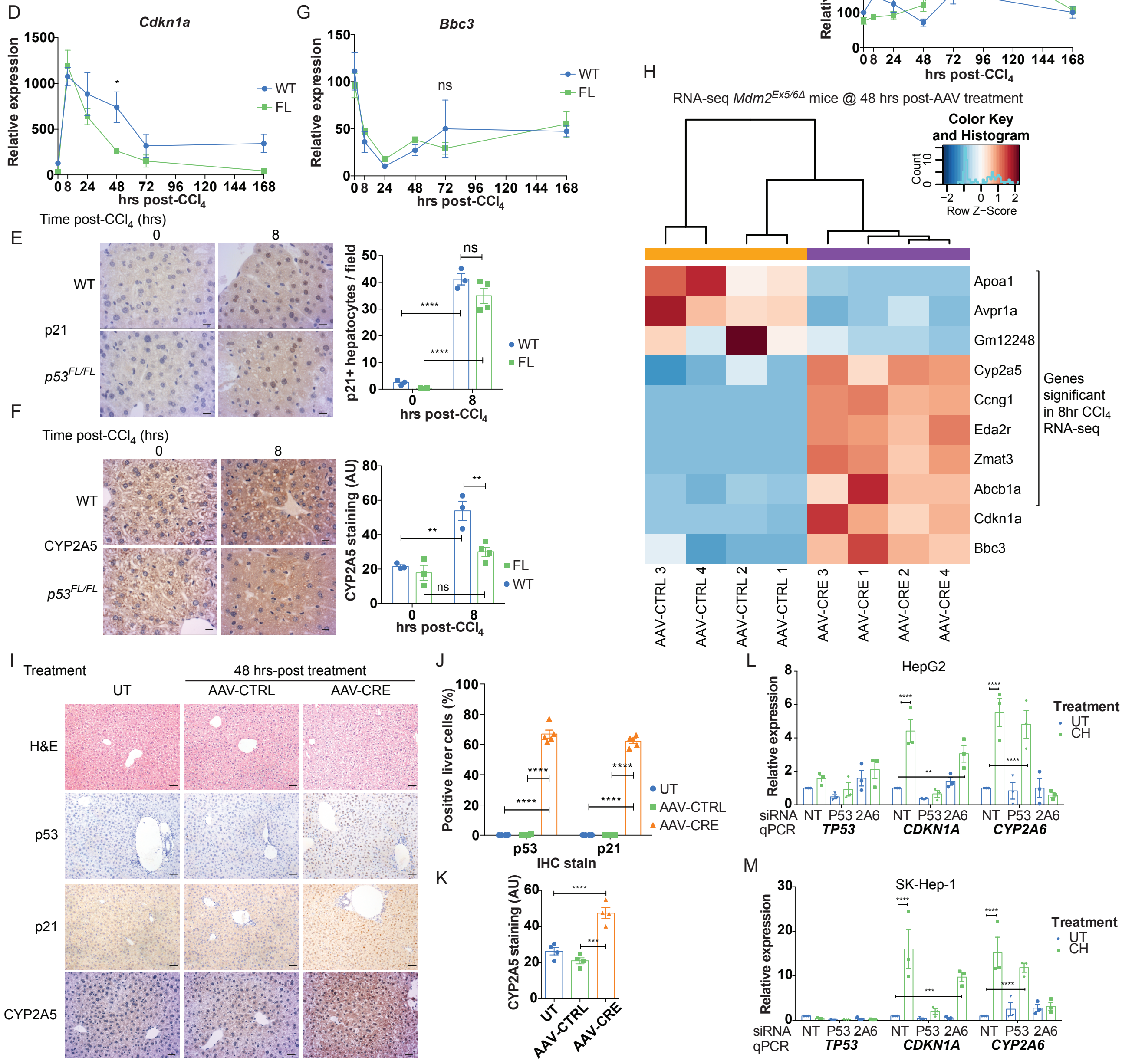

A. Gene list depicting all significant differentially expressed genes (adjusted p<0.05) from RNAseq analysis between *Albumin*-Cre; $p53^{WT/WT}$ (WT) and *Albumin*-Cre; $p53^{FL/FL}$ ($p53^{FL/FL}$) mice at 8 hours after CCl_4 treatment. Samples from N=3 WT and N=4 FL mice included in analysis. Positive fold-change values correspond to genes enriched in livers of *Albumin*-Cre; $p53^{FL/FL}$ mice vs. *Albumin*-Cre; $p53^{WT/WT}$ (WT) mice. For further information, see materials and methods.

B-D. RT-qPCR analysis of expression of *Cyp2a5*, *Nqo1*, and *Cdkn1a* in liver samples from *Albumin*-Cre; $p53^{WT/WT}$ (WT) and *Albumin*-Cre; $p53^{FL/FL}$ ($p53^{FL/FL}$) mice at indicated times (hours) following CCl_4 treatment. N=7 untreated (0 hrs) mice/group, N=3 mice/group at 8hrs, N=4 mice/group at 24hrs, N=4 mice/group at 48hrs, N=3 mice/group at 72 hrs, N=3 mice/group at 168 hrs. Data presented as mean +/- SEM and analysed using two-way ANOVA with Holm-Sidak's multiple comparisons test and multiplicity-adjusted p-values. *p<0.05, ****p<0.0001.

E/F. Representative IHC staining and quantification of p21 positive hepatocytes per field (E) and CYP2A5 staining (F) in high magnification (40X) images of peri-central vein hepatocytes in livers from *Albumin*-Cre; $p53^{WT/WT}$ (WT) and *Albumin*-Cre; $p53^{FL/FL}$ ($p53^{FL/FL}$) mice at 0 (untreated) and 8hrs after CCl_4 treatment. N=3 untreated (0 hrs) mice/group, N=3 WT mice and N=4 FL mice at 8hrs. Scale bars 10μm. Data presented as mean +/- SEM and analysed using two-way ANOVA with Holm-Sidak's multiple comparisons test and multiplicity-adjusted p-values. **p<0.01, ****p<0.0001, ns: not significant.

G. RT-qPCR analysis of expression of *Bbc3* in samples from (B-D). ns: not significant.

H. Clustering analysis of significant differentially expressed genes (adjusted p<0.05) from RNA-seq analysis between $Mdm2^{Ex5/6\Delta}$ mice treated with AAV-TBG-empty control (AAV-CTRL) and AAV-TBG-Cre (AAV-CRE) at 48 hours after injection. Samples from N=4 mice/treatment included in analysis. Positive Z-score values correspond to genes enriched in livers of AAV-CRE treated $Mdm2^{Ex5/6\Delta}$ mice vs. AAV-CTRL treated mice. Significantly differentially expressed genes from CCl_4 RNA-seq (Figure 2C) in brackets and p53 target genes *Cdkn1a* and *Bbc3* shown. For further information, see materials and methods.

I. Representative H&E and IHC staining for p53, p21, and CYP2A5 in $Mdm2^{Ex5/6\Delta}$ mice either untreated (UT) or treated with AAV-TBG-empty control (AAV-CTRL) and AAV-TBG-Cre (AAV-CRE) for 48 hours after injection. N=4 mice/group in UT and AAV-CTRL. N=5 mice/group in AAV-CRE (one of these not used for RNA-seq). Scale bars 20μm.

J/K: Quantification of p53 and p21 positive hepatocytes (%) (J) from mice in $Mdm2^{Ex5/6\Delta}$ mice from (I) and CYP2A5 stain intensity (K) in $Mdm2^{Ex5/6\Delta}$ mice from (H). Data presented as mean +/- SEM and analysed using an ordinary one-way ANOVA with Holm-Sidak's multiple comarisons test and multiplicity-adjusted p-values. ***p<0.001, ****p<0.0001.

L/M. RT-qPCR analysis of expression of *TP53*, *CDKN1A*, and *CYP2A6* relative to *ACTIN* in HepG2 (L) and SK-Hep-1 (M) cells treated with siRNA against *CYP2A6* (2A6) or non-targeting control (NT) 96 hours prior to analysis and additionally treated with either DMSO control (UT) or with cumene hydroperoxide (10μM) (CH) for 24 hours prior to analysis. N=3 independent samples/condition. Data presented as mean +/- SEM and analysed using 2-way ANOVA with Holm-Sidak's multiple comparisons test and multiplicity-adjusted p-values. **p<0.01, ***p<0.001, ****p<0.0001.

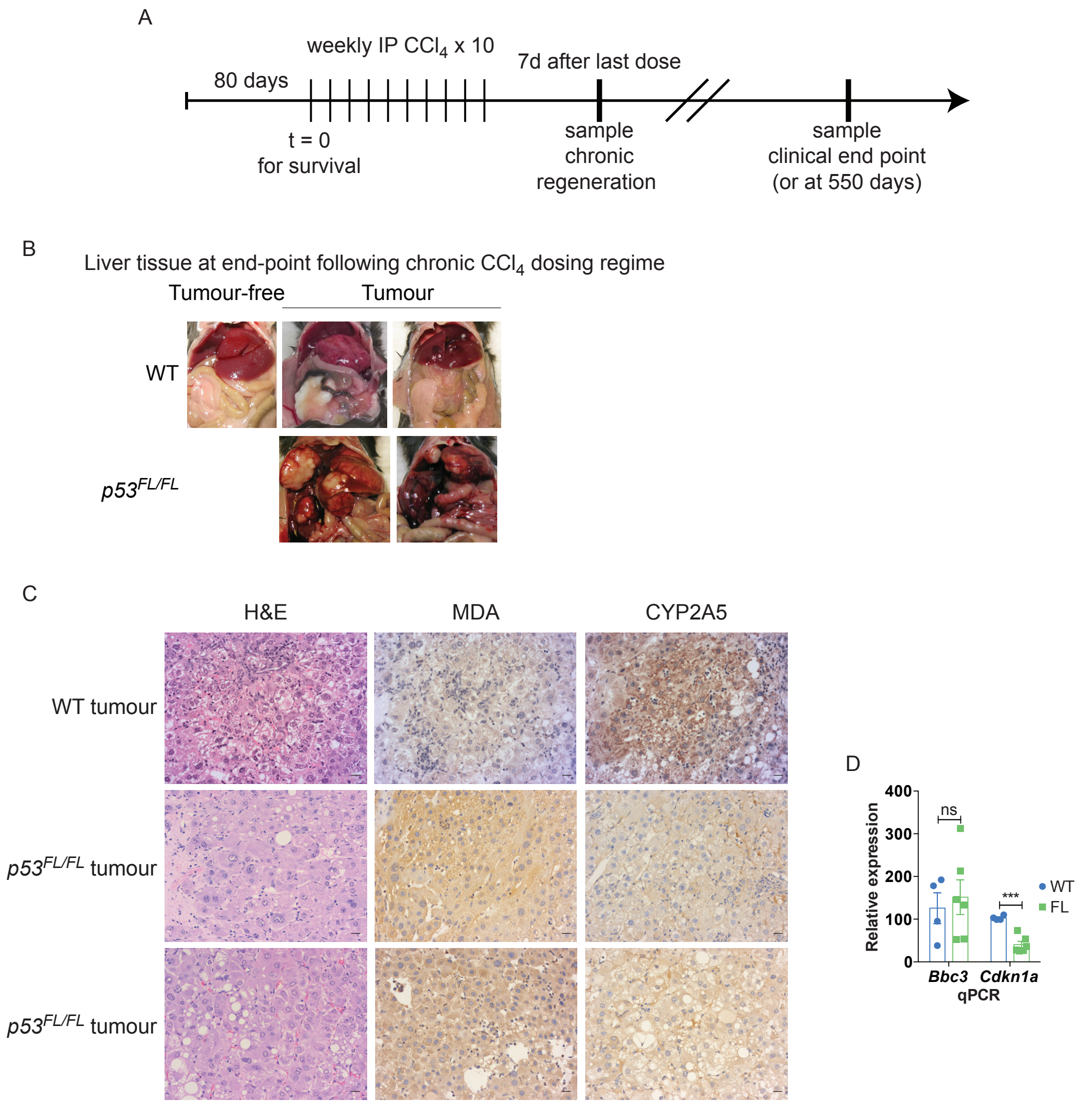

A. Dosing and sampling scheme for CCl_4-mediated chronic regeneration model.

B. Necropsy photographs of liver tissue taken from *Albumin*-Cre; *p53$^{WT/WT}$* mice (WT) and *Albumin*-Cre; *p53$^{FL/FL}$* mice at clinical endpoint (tumour samples) or 550 days post-treatment initiation (tumour-free sample) after prior completion of 10 week CCl_4 chronic regeneration regime.

C. H&E and IHC staining for MDA and CYP2A5 in tumour tissue from *Albumin*-Cre; *p53$^{WT/WT}$* mice (WT) and *Albumin*-Cre; *p53$^{FL/FL}$* mice at clinical endpoint or 550 days post-treatment initiation after prior completion of 10 week CCl_4 chronic regeneration regime. Scale bars 20µm. Additional tumour images shown here illustrate staining from the diversity of tumours observed in the model and supplement those images presented in Figure 4 C.

D. RT-qPCR analysis of expression of *Bbc3* and *Cdkn1a* relative to *Beta-glucuronidase* in tumour samples from *Albumin*-Cre; *p53$^{WT/WT}$* mice (WT) and *Albumin*-Cre; *p53$^{FL/FL}$* (FL) mice at clinical endpoint or 550 days post-treatment initiation after prior completion of 10 week CCl_4 chronic regeneration regime. N=4 WT and N=6 FL samples. Data presented as mean +/- SEM and

Supplemental Table 1: IHC and staining reagents

Reagent	Company	Code
Antibody diluent	Leica	AR9551
BOND Epitope Retrieval Solution 2 (ER2)	Leica	AR9640
Liquid DAB	Agilent	K3468
Rabbit EnVision	Agilent	K4003
Mouse EnVision	Agilent	K4001
Haematoxylin Z	CellPath	RBA-4201-00A
Antigen unmasking solution (pH6)	Vector Labs	H-3300
BLOXALL blocking solution	Vector Labs	SP-6000
Vectastain ABC elite kit	Vector Labs	PK-6010
ImmPACT DAB	Vector Labs	SK-4105
Mouse on mouse blocking reagent	Vector Labs	MKB2212
Peroxidase block	Agilent	S2022
Wash buffer	Agilent	K8007
Direct Red 80	Sigma Aldrich	365548-25G
Fast Green	Raymond Lamb	2008/10/21
Aqueous Picric Acid	VWR	84512.260
Oil-red-O solution	Merck Life Science	1052300025
Mayers Haematoxylin Solution	Sigma Aldrich	51275
Ultramount Aqueous Mounting Medium	Dako	S1964
DPX mountant	CellPath	SEA-1304-00A
Enz 1 antigen retrieval	Leica	AR9551
ImmPRESS HRP goat anti-rat IgG kit	Vector Labs	MP-7444-15

Supplemental Table 2: Primary antibodies used in IHC

Antibody / Stain	Company	Code	Autostainer	Retrieval	Dilution
Glutamine Synthetase	Sigma Aldrich	HPA0007316	Dako Link 48	Vector pH6	1:600
aSMA	Sigma Aldrich	A2547	Dako Link 48	Vector pH6	1:25K
Phospho-H2A.X	CST	9718	Leica Bond Rx	ER2	1:120
CYP2A6 (OTI1D2)	Invitrogen	MA525758	manual	Vector pH6	1:333
MDA	Abcam	ab6463	manual	Vector pH6	1:333
p21	Abcam	ab107099	Leica Bond Rx	ER2	1:150
p53	Leica	NCL-L-p53-CM5p	Dako Link 48	TRS High pH	1:750
NIMP	Abcam	ab2557	Leica Bond Rx	Enz 1	1:600

Table 3: Quantitative RT-PCR primers

Gene	Assay ID
Gusb	Mm00446953_m1
Cdkn1a	Mm04205640_g1
Cyp2a5	Mm00487248_g1
Nqo1	Mm01253561_m1
Bbc3	Mm00519268_m1
ACTB	Hs01060665_g1
CDKN1A	Hs00355782_m1
TP53	Hs01034249_m1
CYP2A6	Hs00868409_s1
